# Barriers to and facilitators of interventions to counter publication bias: thematic analysis of scholarly articles and stakeholder interviews

**DOI:** 10.1186/s12913-014-0551-z

**Published:** 2014-11-13

**Authors:** Christina Kien, Barbara Nußbaumer, Kylie J Thaler, Ursula Griebler, Megan G Van Noord, Petra Wagner, Gerald Gartlehner, on behalf of the UNCOVER Project Consortium

**Affiliations:** Department for Evidence-based Medicine and Clinical Epidemiology, Danube University Krems, Dr.-Karl-Dorrek Str. 30, Krems an der Donau, 3500 Austria; Medical Center Library & Archives, Duke University, Durham, NC USA; Research, Technology & Innovation Policy, Innovation Systems Department, AIT Austrian Institute of Technology GmbH, Wien, Austria; Research Triangle Institute International, Research Triangle Park, Durham, NC USA

**Keywords:** Publication bias, Randomized controlled trials as topic, Trial registration, Access to information, Peer review, Medical errors/prevention & control

## Abstract

**Background:**

When the nature and direction of research results affect their chances of publication, a distortion of the evidence base – termed publication bias – results. Despite considerable recent efforts to implement measures to reduce the non-publication of trials, publication bias is still a major problem in medical research. The objective of our study was to identify barriers to and facilitators of interventions to prevent or reduce publication bias.

**Methods:**

We systematically reviewed the scholarly literature and extracted data from articles. Further, we performed semi-structured interviews with stakeholders. We performed an inductive thematic analysis to identify barriers to and facilitators of interventions to counter publication bias.

**Results:**

The systematic review identified 39 articles. Thirty-four of 89 invited interview partners agreed to be interviewed. We clustered interventions into four categories: prospective trial registration, incentives for reporting in peer-reviewed journals or research reports, public availability of individual patient-level data, and peer-review/editorial processes. Barriers we identified included economic and personal interests, lack of financial resources for a global comprehensive trial registry, and different legal systems. Facilitators identified included: raising awareness of the effects of publication bias, providing incentives to make data publically available, and implementing laws to enforce prospective registration and reporting of clinical trial results.

**Conclusions:**

Publication bias is a complex problem that reflects the complex system in which it occurs. The cooperation amongst stakeholders to increase public awareness of the problem, better tailoring of incentives to publish, and ultimately legislative regulations have the greatest potential for reducing publication bias.

**Electronic supplementary material:**

The online version of this article (doi:10.1186/s12913-014-0551-z) contains supplementary material, which is available to authorized users.

## Background

The non-publication of clinical trial results may lead to false conclusions about the balance of benefits and harms of medical interventions and, in turn, harm patients and waste resources [1]. Publication bias occurs when the publication of research depends on the nature and direction of the results – meaning that a study’s positive, negative, or null result can alter its chances of publication [2,3]. As awareness has increased about the detrimental effects of publication bias, the research community and the public have tried to implement preventive measures such as trial registries, changes in the peer review process, providing access to trial data, and others to reduce the non-publication of clinical trials [3]. According to a systematic review, however, no evidence currently exists that any of these interventions are successful in dealing with the problem [4]. Furthermore, a recent study reported that of 585 registered trials in *ClinicalTrials.gov* which had been completed prior to January 2009 133 (23%) provided no results either in published form or in *ClinicalTrials.gov* [5]. This means that an estimated number of 254,000 patients participated in trials and placed themselves at risk for studies that never became public knowledge [5]. A systematic review on the extent of publication bias showed that the total number of studies not published in each investigated subgroup (e.g. study protocols approved by ethics committees or funding granted by research funders) varies widely from 7% to 79%. Furthermore, 31% to 62% of studies had at least one primary outcome that was changed between study registration and publication of results [6,7].

Why measures against publication bias fail remains unclear. Several stakeholder groups with partially competing interest are involved in the process of funding, conducting, and publishing research (e.g. researchers, publishers, sponsors, regulatory agencies). These groups of stakeholders have different motivations and interests to publish or not publish research. Information about barriers and facilitators for measures intended to reduce the non-publication of trials from the perspective of these stakeholders would be valuable for designing and tailoring future interventions.

The objective of our project was to use qualitative techniques to determine factors that can act as barriers or serve as facilitators in the implementation of interventions to prevent or reduce publication bias resulting from non-publication of clinical trials. We focused on the non-publication of entire clinical trials which needs to be distinguished from selective outcome reporting which occurs if only a selection, on the basis of the results, of a subset of analyses is to be reported [8].

## Methods

To determine factors that can act as barriers to or serve as facilitators of interventions to prevent or reduce publication bias, we employed two qualitative approaches. First, we qualitatively analyzed scholarly articles; second, we conducted interviews with stakeholders. We chose this approach to gain a broad overview of opinions of stakeholders, including those that might be underrepresented in journal articles (e.g. funders, ethics committees). In addition, we tried to identify and include ideas and trends which have emerged after the literature search for this review. The study was registered with the Austrian Data Protection Authority (number: 4008646) and performed in accordance with data storage and confidentiality regulations. We didn’t seek ethical approval at a research ethics committee as the study did not involve medical research. The methodology of the interviews adheres to the RATS guidelines for qualitative research [9]. In the following sections we describe the methods of both approaches in more detail.

### Review of scholarly articles

The basis of the analysis of scholarly articles was a systematic review. We searched MEDLINE (via PubMed), the Cochrane Library, EMBASE, CINAHL, PsycINFO, AMED, and Web of Science up to May 2012. We used Medical Subject Headings (MeSH) and free text key words for publication bias and other related biases, as well as for interventions used to reduce the non-publication of trials based on Song et al. [3]. We searched for publication and all related biases (because this search strategy was also used for a systematic review on the effectiveness of interventions with a broader scope [4]), so that we could identify relevant articles on well-known as well as new interventions to prevent publication bias (Additional file 1). We didn’t consider interventions such as disclosure of conflict of interest or large confirmatory scale trials because these are more relevant for detecting or reducing other forms of biases such as selective outcome reporting bias. In addition to electronic searches, we manually searched reference lists of pertinent reviews and articles.

Two trained research team members independently reviewed all titles and abstracts identified through searches. We retrieved and reviewed the full text of all titles included during the abstract review phase. If both reviewers agreed that an article did not meet the eligibility criteria, the study was excluded. If the reviewers disagreed, conflicts were resolved by discussion and consensus or by consulting a third member of the review team. We tracked all results of the literature review in an EndNote® X6 bibliographic database (Thomson Reuters, New York, NY) [10].

One researcher extracted relevant text passages (i.e., type of article, intervention, the mentioned barrier or facilitator and context information), a second checked correctness. We included different types of scholarly articles in order to identify expert opinions and common themes about facilitators and barriers: any empirical research studies, such as expert interviews or surveys that examined barriers and facilitators in the implementation of a measure to reduce publication bias, narrative literature reviews, editorials, commentaries, and letters to the editor. We did not perform a risk of bias assessment because our aim was to give an overview of possible barriers and facilitators of the implementation of interventions to counter publication bias, not their effectiveness.

For the analysis of the text passages from the included articles we performed an inductive thematic analysis as described by Braun & Clarke [11]. A thematic analysis is a qualitative method to identify, analyse, and report themes by searching across a data set – in our case a range of texts. The first step in the analysis was to assign initial codes to the text. Similar codes were then combined into themes. Finally, themes were clustered into higher-ranking themes (i.e. main categories subsuming descriptive themes). The themes were identified inductively from the data and were not predetermined before we started the analysis. We used the software MAXQDA Version 10 [12] to support the coding process.

### Stakeholder interviews

We conducted semi-structured interviews [13] with representatives of relevant stakeholder groups. We defined relevant stakeholders as any group or individual that can affect or is affected by the publication of a clinical trial, such as ethics committees, patient organisations, the pharmaceutical industry, political decision makers, journal editors or associations, regulatory agencies and supporting organisations (e.g. trial registries), research funding bodies and research institutions and associations. We determined relevant stakeholder groups by developing a stakeholder map [14]. In general, organisations were the unit of research because they are thought to have a multiplying effect in either gathering the perspectives of or in broadening ideas to member institutions or individuals. The focus was on organisations on the European level (regarding ethics committees, funding or research organisations) and extended to organisations acting worldwide (e.g. trial registries, professional organizations) to include international influences. As interview partners we invited persons involved in the implementation of new policies or representatives of relevant European stakeholder groups. To minimise selection bias, two researchers discussed the ongoing invitation process. We identified potential interview partners through literature included in the thematic analysis of articles, extensive internet searches and known experts of the field. Overall, we used purposive sampling (i.e. selecting the interviewees who would be most likely to contribute relevant and in-depth data) [15].

Two researchers developed an interview guide which focused on questions about reasons for publication bias and barriers and facilitators regarding interventions to counter publication bias in general, as well as in relation to the specific organisation. Four experienced qualitative researchers led interviews and adapted the interview guide (Additional file 2) during the process as needed to enable a fluent interview (semi-structured interview) [16]. We gathered data mainly by telephone or via Skype™ (Version 6.9.0.106) without video and we also conducted face-to-face interviews. In two cases we accepted responses to the questions in writing because of scheduling difficulties.

We asked for participant permission to record and transcribe interviews while maintaining anonymity. All interviewees provided informed consent. Interviews lasted between 20 to 50 minutes. We applied the same thematic analysis approach as described above in the section on scholarly articles [11]. Two coders assigned initial codes to the text. Similar codes were then combined into themes drawing on themes from the thematic analysis of the articles as a starting point. As new codes were identified in the analysis of the interviews, we generated new themes accordingly. To ensure that ambiguity in the coding and analysis process were reduced, quality assurance measures were taken: After one coder finished assigning codes to the first interview, the results were discussed and changed if necessary. This process was repeated with the second coder’s first interview. A discussion process between two coders was set up to foster the understanding of the data and to solve ambiguities in establishing themes [17,18].

## Results

Before presenting the combined results of the scholarly literature review and the interviews, we describe the identified literature and the sample of interviewees.

**Figure 1:**
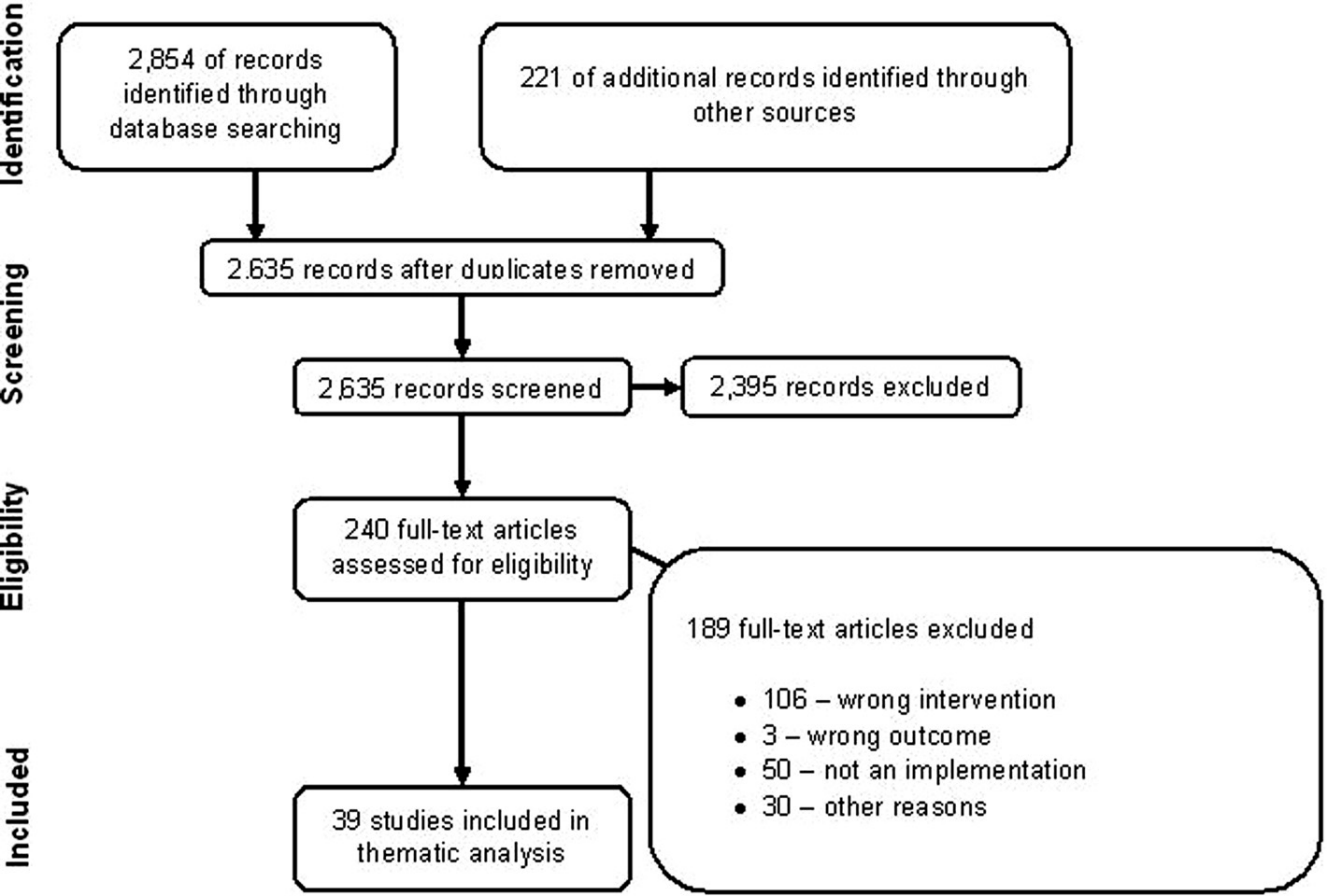
**PRISMA: disposition of the articles.**

Of 2,635 records screened, we included 39 articles for the thematic analysis of scholarly articles (Figure 1). We found one empirical research study (web-based survey) on researcher opinions on registering trial details [19] and two empirical research studies on the peer review process [20,21]. We also included six research studies that mentioned barriers/facilitators of peer review, and prospective trial registration in the discussion part of the articles [22-27]. We identified one article discussing an explanatory framework of factors influencing peer review [28]. We found six narrative literature reviews [29-34], eleven commentaries [35-45], seven editorials [46-52], three letters to the editor [53-55], and two articles that described specific trial registries [56,57]. Some articles mentioned barriers and/or facilitators for more than one intervention to counter publication bias (Additional file 3).

In addition, we invited 89 stakeholders, of which 55 either did not reply after a reminder or refused to be interviewed (reasons: no time, no expertise on this topic, organisation’s communication policy, refusing to share information on this research topic). Thirty-four stakeholders (response rate: 38%) from nine different stakeholder groups agreed to be personally interviewed in the time period between November 2012 and May 2013 (Table 1). Thirty interviewees represented European or European-based organisations, four interviewees represented international organisations. The response rate was similar to other studies in this field (ranging from 22% [19] to 48% [58]). We achieved saturation as we observed the recurrence of themes between interviewees (i.e. no new items appeared in the interviews). Further, we completed interviews with associations and networks on the European level for each stakeholder group. Because we were unable to interview political decision makers, regulatory agencies and the pharmaceutical industry, we consulted three policy documents relevant to these groups [59-61]. We analysed 37 documents in addition to the 39 scholarly articles.

**Table 1:** **Invited interview partners and conducted interviews**

**Groups of stakeholders**	**Invited**	**Conducted**	**Policy documents**
Ethics committees	19	4	-
Patient organizations	6	1	-
Pharmaceutical industry	9	2	1
Political decision makers	2	1	1
Publishing (journal editors, associations)	9	4	-
Regulatory agencies and supporting organizations (e.g. trial registries)	15	6	1
Research funding bodies	14	5	-
Research institutions and associations	10	6	-
Other groups: networks, associations	5	5	-
**Overall**	**89**	**34**	**3**

In the following section we provide a synthesis of results from the review of articles and stakeholder interviews. We arrived at four interventions to counter publication bias based on Song et al. [3] and categorized the information into barriers and facilitators according to the following interventions: prospective trial registration, incentives for reporting results in peer-reviewed journals or reports, policies regarding public availability of individual patient-level data and the peer-review and editorial process.

We mark barriers and facilitators in italic font. Verbatim quotes from interviewees are presented with quotation marks. Table 2 provides an overview of identified barriers to and facilitators of interventions to counter publication bias.

**Table 2:** **Identified barriers and facilitators for possible interventions to counter publication bias**

**Barriers**	**Facilitators**
**Prospective trial registration**	
• Competing economic or personal interests of different stakeholders	• Trial registration as a prerequisite for crucial decisions within research (e.g. approval from ethics committees, publication by journal editors, condition of funding)
• Lack of mechanism to enforce trial registration	• One comprehensive trial registry
• Lack of awareness of the problem	• One unique registration number
• Imperfect data quality (e.g. incomplete data entries)	• Provision of resources to maintain trial registries
• Lack of sufficient resources to enable registries to improve data quality	• Raising awareness
• Many trial registries with different purposes exist	• Educating stakeholders
• Different legal systems in different countries	• Support of all stakeholders
**Incentives for reporting in peer-reviewed journals or research reports**	
• Lack of prestige for publishing negative findings	• Guidelines for Good Clinical Practice
• Perceived lack of possibilities to publish	• Right to publication
	• Monitoring of publication status by ethics committees via providing a route to maintain a track record
	• Retaining a certain percentage of the research grant until results have been published by funders
	• Law requiring publication of results
**Public availability of individual patient level data (IPD)**	
• Competing financial or career related interests	• Incentives for making IPD publically available (reputation and credibility, proliferation and efficiency of health care research, development of a new research evaluation system)
• Safeguarding the privacy of patients	
• Reporting requirements	• Fostering cooperation and exchange between researchers
• Missing quality checks	• Law requiring the (restricted) public availability of IPD
• Complex technological requirements	• Monitoring of complying and mechanisms of enforcement
**Peer-review process and editorial processes**	
• Influenced reviewers and editors	• Enforcing objectivity
• Cultural norms and behaviours	• Disclose of conflict of interest
• Inconsistencies in the process	• Use of professional peer reviewers
• Lack of consistent qualifications	• Training for peer review and editors
	• Peer review only introduction and methods part of a manuscript

### Prospective trial registration

Prospective registration of all clinical trials in a searchable and comprehensive registry can ensure that basic information about all trials is accessible to the public [36]. This can help researchers identify the proportion of unpublished trials. US law requires prospective registration of trials and mandatory reporting of results within one year of trial termination [62]. The European Medicines Agency (EMA) requires researchers to include details of clinical trials concerning medicinal products for human use in the European clinical trials database (EudraCT, established in May 2004) within the authorisation process. Since March 2011, limited information on trial methods – but no results – are publically available through the EU Clinical Trial Registry (EU CTR) [63]. The law does not require the inclusion of other interventions such as studies concerning medicinal devices or other non-drug-trials. Information contained in registries is often incomplete [6,7].

### Barriers

Authors discussed *economic and personal interests* as major barriers to trial registration. Pharmaceutical companies pursue commercial interests and fear that financial losses could occur with the registration of trials [19,24,33,52,57]. They believe that publication of information on new developments could threaten confidentiality and lead to competitive disadvantages [31,33,35,51]. Academic researchers also have competing interests, such as the right for exclusivity on research ideas [19,33,51]. Although the US and EU have adopted laws requiring trial registration as well as the reporting of results, the US and EU have not implemented *monitoring or mechanisms of enforcement* [29,31,36,43,50]. A *lack of awareness* of the problem and the consequences of publication bias was identified as a barrier [31]. In identifying the shortcomings of trial registries, several articles mentioned the *imperfect quality of data* [26,27,46], as well as the *lack of sufficient resources to enable registries* to check and improve the quality of included data [29,31,52]. Furthermore, although *many trial registries* exist worldwide, they vary greatly in their stages of development, coverage, and goals. In addition, different groups may be in charge [31,50,52,56]. The World Health Organization International Clinical Trials Registry Platform (WHO ICTRP) has defined specific criteria for content, validity, etc. and listed trial registries which meet these criteria [64]. The broad range of registries makes the effort to locate and capture all trials from such a wide variety of sources difficult [36,52]. Any initiative to create a worldwide uniform trial registry would have to conform to the laws of often *vastly different legal systems* [29,50,52].

### Facilitators

*Mechanisms to enforce trial registration* could increase the amount of trials registered and improve the quality of data. One interviewee said: “For registers to be very comprehensive there is a need for sometimes more than just guidelines and recommendations” (Interviewee [I] #9, 61). This opinion was echoed by several other interviewees. As a solution, some authors suggested imposing monetary penalties, withholding future funding, or issuing public notices of noncompliance [25,30,31,33,51]. One mechanism on the regulatory side could be to tie licensing with prospective registration. Only drugs with studies which have been prospectively registered will be considered for licenses (e.g. will be considered in the current Regulation of the European Parliament and of the Council on clinical trials on medicinal products for human use, repealing Directive 2001/20/EC [61] and was considered in the U.S. Test Act [65]).

Both interviewees and authors suggested *that trial registration should be a prerequisite* for crucial decisions within research (e.g. trial approval from ethics committees, decision from research funding bodies) [22,31,33,41,43,50]. Since 2013, trial registration is required as a prerequisite for ethical approval in the UK [66,67]. Registration as a prerequisite for consideration of publication in peer-reviewed journals was implemented in September 2005 by the International Committee of Medical Journal Editors (ICMJE) [68] and resulted in an increase in the number and proportion of clinical trials being prospectively registered by 73% between May and October 2005 from 13,153 to 22,714 [69]. Despite this policy, ICMJE-member journals showed a lack of monitoring and enforcement of this policy [7] and many journals have not implemented this policy at all [24,26,29,33,35,46]. An interviewee recommended that journals should “retake their vows, like people do with marriages sometimes” (I#10, 41).

Overall, having *one comprehensive trial registry* for all trials and using a *unique registration number* would simplify the search for trials [29,31,34,50], and would enable the differentiation between multiple studies and multi-center trials [29,31,36,43]. As interviewees added, transparency could be fostered by linking study protocol, trial registration, and publication of results. Authors recommended *worldwide legislation* that mandates registry of trials and international linked registries [29]. One interviewee highlighted an example where surveillance and control of health problems that do not have international boundaries were tackled at an international level, namely the World Health Organisation (WHO) Framework Convention on Tobacco control [70].

Articles call for governments and the pharmaceutical industry *to provide resources* to maintain trial registries [29,33,43]. In order to guarantee independence from the pharmaceutical industry, an independent fund for trial registries with blind financial support from different sources could be established. Appropriate software to manage such a huge amount of information is described as a necessary resource [29,52]. Further, *better usability* (simplification of the process, improvement of the explanatory text including requirement for registration) would foster the uptake and the quality of entries in trial registries [26,29,31].

To increase the prospective registration of studies, initiatives should focus on *raising awareness*, as well as educating and gaining the *support of stakeholders* (research funders, sponsors of trials, governments, journal editors) [31,33,48]. Representatives of trial registries, funders, ethics committees, and journal editors pointed out that an important part of their work is to inform researchers about the necessity of registering trials and properly reporting clinical trial results. Another interviewee mentioned the need to address the consequences of publication bias in the curriculum of researchers, stating “I think that’s a thing where those [of us who] have been involved in teaching medical students have probably not been as effective as we should have been. And I think that’s because the extent of the problem of publication bias is something that we ourselves have not really fully taken on board*.”* (I#15, 36). Later on the interviewee added that he became only fully aware of the consequences of publication bias via the “All Trials Registered – All Trials Reported Campaign” (www.alltrials.net) which was set up in January 2013 [71].

### Incentives for reporting in peer-reviewed journals or research reports

The proportion of published trials also depends on the motivation of investigators to submit results for publication to peer-reviewed journals [72]. Half of European Union health-related funded studies between 1998 and 2006 were not identifiably published [73]. The following section addresses barriers and facilitators on incentives and regulation for reporting in peer-reviewed journals or research reports.

### Barriers

An important identified barrier in the interviews was a *lack of prestige* for publishing *negative results*. One interviewee said: “Clearly that’s not as prestigious nor is it interesting and [researchers] don’t get cited […]. You do not go to a [grant] body with a negative result.” (I#8, 14). Research shows that positive results are cited more often than negative results, and the impact factor calculation of journals depends on citations [74,75]. Currently, the impact factor is used as a decision base for career advancement for researchers and grant awards [76].

Another barrier was a *perceived lack of possibilities to publish*. Several interviewees argued that in accordance with “the freedom of the press,” journals should continue to be able to select what they want to publish. Although several journals do exist for the sole purpose of publishing indeterminate or null findings, these must not be so well-known since several interviewees, in particular, researchers, suggested that such journals should be created. The interviewees as well as authors argued that *public funding* could separate the publication process from financial constraints and therefore facilitate the existence of such journals [47].

### Facilitators

Authors and interviewees suggested different ways to foster the reporting of results. Several authors argued that it is an ethical responsibility to share all results [35]. A similar vein is followed by *Guidelines for Good Clinical Practice*, such as The Declaration of Helsinki (DoH) issued by the World Medical Association [77] and The European Code of Conduct for Research Integrity [78]. They are relevant to publication bias as both the DoH [77] and the European Code of Conduct [78] explicitly mention that research results have to be published. In general, interviewees considered the Code of Conduct as “theory” and viewed funding bodies as having the necessary leverage to ensure that research institutions accept and implement these guidelines. Interviewees recommended that the DoH and the Code of Conduct should be an integral part of PhD-programs and could be promoted further through talks and international seminars. Overall, interviewees admitted, that there is no ideal process to disseminate guidelines due to legal and cultural differences. Interviewees also highlighted the necessity of insisting on a contractual *right to publication* of research results when cooperating with sponsors.

Many authors considered *ethics committees* to be in the ideal position to enforce the dissemination of trial results; they could require reports and send them to a central, comprehensive, and multidisciplinary registry which would have to be set up [41,54,55]. If ethics committees take on this role, it will be important that they receive appropriate resources [50]. One interviewee suggested that this could be fostered by providing an automatic route to maintain a track record of transparency. Interviewees from European countries other than the UK explained that research ethics committees must be harmonized nationally and across Europe to enable this role (e.g. standardized operating procedures, a well-functioning application system, on-going training of ethics committees members, and quality assurance measures).

*Funding bodies* could also ensure that the results of financed trials are publicly disseminated [47,50]. Several European funding bodies have policies regarding the publication of trial results in open access journals [79] but these policies are not always mandatory or include no enforcement mechanisms. Interviewees suggested that only research organisations with publication policies should receive funding in order to transfer policies and norms from funding organisations to research organisations. In a further step, interviewees requested that funding bodies put sanctions in place for failure to publish, such as retaining a certain percentage of the research grant until results have been published. For this to happen, interviewees mentioned that the following points need to be taken into consideration: First, sanctions can be implemented only if they adhere to national laws. Second, national funding bodies often consist of a broad range of funders, meaning that proposed sanctions might be subject to an extensive approval process.

### Public availability of individual patient-level data

Although trial registration is important for detecting publication bias, several authors and interviewees argued that another great need exists for full availability of individual patient-level data (IPD) of clinical trials: “We cannot rely on what people make available in tables, we already know that. So we also need access to the raw data, so that there can be an independent assessment of the same data” (I#1, 22). In November 2012, the EMA stated that they are committed to the “proactive publication of data from clinical trials supporting the authorisation of medicines once the marketing-authorisation process has ended, which the EMA does not consider commercially confidential” [80]. The EMA issued a draft policy on access to clinical trial data [60], and declared that it would not proactively publish clinical trial data but would “give the possibility to download, save and print the trial data for academic and non-commercial research purposes” by October 2014 [81]. Some interviewees argue that the publication of summary results is sufficient, especially, as several barriers remain.

### Barriers

University and pharmaceutical industry researchers may have *competing interests* when it comes to publishing (or not publishing) IPD due to career or financial concerns. Researchers may be reluctant to publish results in a registry before publication in peer-reviewed journals, even if the journals would accept such pre-publication [19]. Interviewees stated that researchers usually want to publish several articles based on one data set because randomised controlled trials (RCT) tend to be very resource and time intensive. Several interviewees mentioned concerns over authorship and recognition for data providers when others conduct subsequent analyses on data provided in registries. Pharmaceutical companies are especially concerned about losing their competitive advantage, if “trade secrets and proprietary information” [59] were made publically available. Several interviewees mentioned the need to safeguard the *privacy of patients* if IPD were made publically available, especially regarding socially stigmatized or rare diseases. Special concerns were raised regarding misuse or manipulation which could occur through linkage of data with other data carriers [60]. Some interviewees argued that anonymisation strategies may not be full proof. If the protection of patient privacy is to remain a primary goal, then the small risk that individual patients could be identified must be weighed against the overall public health gains.

Interviewees and authors argued that solutions for *reporting requirements* are needed [82,83]. Study details such as randomisation and allocation concealment procedures and intervention descriptions should be provided so that, “data aren’t just publically available but publically understandable” (I#4, 106). For data submitted to the EMA, the required standard will be the Clinical Data Interchange Standards Consortium (www.cdisc.org) [60]. One interviewee argued that for IPD reporting, open standards that are publically available and subject to discussions and change, should be applied to avoid lengthy reporting standard discussions.

Another barrier interviews and articles identified to the usage of data repositories was *missing quality checks* of publically available data as well as planned secondary analyses [40]. To store and search for IPD, *technological systems* are required that are more complex than those needed for the storage of pdf-formatted articles. Interviewees mentioned several examples of such registries, such as the Dataverse Network™ Project (http://thedata.org) [84].

### Facilitators

Interviewees mentioned the following *incentives* for making IPD publically available. In contrast to concerns raised by pharmaceutical companies regarding competitive advantage losses, interviewees highlighted gains in reputation and credibility due to improved transparency. They mentioned increased efficiency in health care research due to aspects such as improvements in comparative-effectiveness analyses and validation of outcome assessments [39,45]. Interviewees reasoned that given recent cases of scientific misconduct in the academic field, academia would also benefit. With regards to the role of impact factors in the academic world, one interviewee explained how research funders imagined a different evaluation system of conducted research: “When [research funders] evaluate projects [they should] not just look at publication lists of the principal investigator, but also at researcher’s record in storing and managing data, in publishing open access and not only in high impact journals” (I#11, 36). Furthermore, one interviewee added, that *fostering cooperation and exchange* between researchers would be an important basis for sharing data. Journal editors also have a stake in credible and transparent research, and therefore support open access to data [85].

Several interviewees who support public availability of IPD highlighted the need for a *law which requires the publication of results, and IPD in particular,* which is in line with data protection requirements [45]. Preferably, an interviewee reasoned, cooperation would occur between different nations with the goal of passing similar laws to exert a common effect on industry or individual researchers and prevent the pharmaceutical companies’ drift to Non-European countries. Again, *monitoring* of compliance with a law and different *mechanisms of enforcement* were discussed in both the interviews and the articles (e.g. financial sanctions [45], posting non-compliance on professional or patient organisations websites, including statements in Cochrane Reviews regarding the amount of data of clinical trials could not be considered in the assessment of a certain treatment due to public unavailability).

### Changes in peer review and editorial processes

Journal peer review has been defined as “the assessment by experts (peers) of material submitted for publication in scientific and technical periodicals” [86] aiming to improve the general quality of a study [3].

### Barriers

Peer review is a highly subjective process; personal knowledge [28], private interests of reviewers such as professional affinities or rivalries [21,42], a preference towards interesting topics and favourable results [23], or towards the confirmation of their own thinking [28,49] can affect the review (i.e. *biased reviewers*)*.* Similarly, editors can be influenced by their personal beliefs and attitudes (i.e. *biased editors*) [21,28]. Both authors and interviewees argued that manuscripts of studies with failed treatments are less likely to be cited which can influence the impact factor of the journal and may therefore lead to rejection by the editors [20,38]. One interviewee added that editors also tend to reject manuscripts that are industry-funded. Several interviewees pointed out that the perceived barrier of biased reviewers and editors may prevent authors from pursuing publication. Some admit that while publication of negative results may be possible, it is less likely in the most prestigious journals. This results in an imbalance between the work required to publish and career advancement. An interviewee gave the following example: “Because journals want something, which […] will be quoted often, [… and] will make some kind of impact, […] saying that something does not work […], may end up, in a [non-English] journal and not in a good international one” (I#20, 18). Further, another interviewee stated, “I think research does suffer, so, so we do need to change that perception” (I#6, 73).

Furthermore, we identified *inconsistencies in the peer review process* as one perceived barrier [21,42]. For instance, codes of practice among medical journal editors are voluntary and do not appear to be common [28]. Consistent criteria may be lacking for the selection of peer reviewers [23,40] and manuscripts [21,37]. Although training manuals such as from the Committee of Publication Ethics (COPE) or European Association of Science Editors (EASE) for editors and peer reviewers are available, they do not seem to enjoy widespread use [20,42].

### Facilitators

The articles discuss several ways to overcome inconsistencies in the peer review process: *enforcement of transparency* through publication of abstracts along with an explanation for rejections [49]; e*nforcement of objectivity* through incorporation of opinions from a wide range of experts [49] or implementation of an agreed upon quantitative measurement in the peer review process, familiar to all manuscript authors [37]; the inclusion of a *statement of conflict of interest for reviewers and editors* to identify personal relationships, academic rivalries, or personal, political, or ideological persuasions were proposed [21,49]; usage of *fulltime, experienced professional peer reviewers and editorial boards* to facilitate proper implementation of the peer review process [42] and *the introduction of training for peer reviewers and editors* in scientific appraisal of the submitted manuscripts [21,42,49] as well as the *raising of awareness* that manuscripts with neutral or negative results are of interest and value to the scientific community and study quality, not just positive outcomes, should be the focus [20,23,32]. Other proposals focused on the *parts of a manuscript which should be subject to peer review* to prevent reviewers from being biased by the results of the study: only the introduction and methods sections, everything except the results [53] or the peer review of the protocol. If it’s determined to be good, the journal commits to at least sending the manuscript of the study out for peer-review. This way an editorial commitment is made before the results are known [44].

## Discussion

To our knowledge, our study is the first to provide a comprehensive analysis of barriers and facilitators of the publication of trial results as discussed in published literature and gained from interviews with stakeholders. Our approach enabled us to verify the results gained from the literature review, to use interviews to put results of the literature review into perspective and identify new insights not addressed in the literature. The most common barriers to trial publication included economic or personal interests, lack of sufficient funding for a comprehensive trial registry, differing legal regulations, and concerns over patient privacy. Important facilitators were incentives for the publication of statistically non-significant results (e.g. research funders adopt performance metrics including full dissemination of research and providing data sets), and laws for the enforcement of prospective registration and reporting of clinical trial results. A common theme in our analyses is the necessity of an increase in public and researchers’ awareness of the detrimental effects of publication bias on health care. A second overarching theme was that publication bias is a complex problem that demands mutual support within and across stakeholder groups. Such a manifold approach has also been recently highlighted [87].

Our study has several limitations. First, because of the lack of empirical data on barriers and facilitators for the publication of trials, we had to focus on editorials and discussions that expressed the personal opinions of authors active in the academic and regulatory field. Interviews provided the opportunity to integrate perspectives of different stakeholders such as funders, ethics committees, and industry representatives that are not as well represented in the scientific literature as researchers (e.g. role of funding bodies in the implementation of the Code of Conduct in European research organizations, needed harmonization of ethics committees within a country but also across Europe to apply to the role of monitoring publication status).

Second, our literature search covered only the time period up to May 2012. Many new articles on publication bias have been published since then. However, we believe that we captured more recent trends in interviews, conducted almost one year later (e.g. AllTrials Initiative and the associated discussion of making IPD publically available, direct interaction of trial registries with funding bodies).

Third, we conducted interviews with all relevant stakeholder groups and consulted policy documents when stakeholders declined to be interviewed. Because each interviewee was an expert in a specific area, not all questions relating to publication bias could be posed to every interviewee. However, recurring themes across interviews suggest that the number of interviews was sufficient. Overall, the topic of publication bias has been a hotly debated subject in recent years. Although we have conducted a thorough literature review and a purposive sampling of interviewees, we may have missed some barriers and facilitators (e.g. time and personnel costs for making IPD publically available). Furthermore, two online portals were started in January 2014 by the pharmaceutical industry to gain access to results of clinical trials to researchers [88,89], therefore not reflected in the results of our interviews or literature review.

Our study focuses on the European situation and the specific role of certain stakeholders (e.g. research funders, ethics committees, research organizations). However, since pharmaceutical companies and academic research activities are subject to the norms of a globalised world, we also discussed barriers and facilitators on a global level (e.g. WHO ICTRP, ICMJE, international laws concerning trial registration) affecting the specific European situation. Nevertheless, we believe that the extent to which results can be generalized beyond the European context is limited, specifically in relation to current barriers and facilitators in trial registration in developing countries [90,91]. Although some interventions may also be relevant for detecting or countering selective outcome reporting bias (e.g. trial registration), the focus of our study was on non-publication of clinical trials. Therefore we did not cover any measures specific to selective outcome reporting bias.

Findings of our study also highlight several general and contextual issues that policy makers need to address to facilitate the publication of trial results. One recurring theme concerned the problems of differing health care and research systems worldwide (e.g. local ethics committees, national funding bodies, etc.). Several initiatives are currently underway to address these challenges and to build a basis for mutual exchange by creating common standards and procedures and promoting collaboration via the exchange of good-practice examples (e.g. fostering exchange on funding requirements, promotion of guidelines on research integrity, research ethics committees, e.g. EUREC [92]). The WHO ICTRP supports the development of common standards and fosters a meta-registry with a unique identifier number [93]. We recommend strengthening these networks (e.g. fostering learning via the publication of good-practice examples) and increasing financial resources (e.g. for the WHO ICTRP by members, i.e. individual states; for European based networks such as EUREC by research programs). A second issue concerns the lack of mandatory reporting of clinical trial data and results. The EU Clinical Trial Regulation, which was implemented in June 2014, but will not come fully into force until May 2016, could become a milestone with respect to transparency of clinical trial data and results [61,94,95]. A third problem repeatedly mentioned in interviews and by academic stakeholders concerned the lack of awareness among researchers with respect to the detrimental effects of publication bias. Efforts should be made to train and educate researchers regarding publication bias and usage of trial registries. These initiatives should include highlighting journals such as the Journal of Pharmaceutical Negative Results, the Journal of Unsolved Questions (JUNQ), and the Journal of Negative Results in BioMedicine that publish indeterminate or null findings. In addition, public pressure is needed to urge lawmakers to pass legislation that requires trial and result reporting and includes effective measures of enforcement. Until legal changes become effective, interviewees suggested that systematic reviewers who are unable to obtain unpublished data [96] and are confronted with publication bias could use systematic reviews and press releases to draw public attention to those who do not publish trial results. Finally, the lack of consequences for not publishing trial results makes existing measures ineffective. Trial registration and reporting of results should be prerequisites for the acceptance of trial results by regulatory agencies. Publication strategies need to become a mandatory aspect for ethics committees. In the future, a priori registration, open access to trial data, and open access publications have to become prerequisites for receiving grant money funded by tax payers.

## Conclusions

Our study used a novel approach in combining a systematic review of articles with qualitative interviews to identify barriers to and facilitators of four interventions to counter publication bias: prospective trial registration, incentives for reporting results in peer-reviewed journals or reports, policies regarding public availability of IPD and changes in the peer-review and editorial process. One important first step to overcome barriers of interventions to counter publication bias is to increase public and stakeholder awareness of the effects of publication bias on health care and patients through education. Furthermore, the cooperation between stakeholders to implement interventions to counter publication bias, using new performance metrics and therefore creating new incentives for researchers to disseminate their results and data and ultimately legislative regulations have the greatest potential for reducing publication bias.

## Additional files

Additional file 1:
**Literature searches.**
Additional file 2:
**Template for the tentative interview guide.**
Additional file 3:
**Types of articles and interventions addressed in thematic analysis for articles.**
