# Supplementary material for: Barriers to and facilitators of interventions to counter publication bias: thematic analysis of scholarly articles and stakeholder interviews
Source: BMC Health Serv Res. 2014 Nov 13;14:551. doi: 10.1186/s12913-014-0551-z (PMC4310031; doi:10.1186/s12913-014-0551-z)
Supplement: Additional file 1: — Literature searches. [file 12913_2014_551_MOESM1_ESM.docx]

Additional file 1: Search strategy

PubMed

| **Search** | **Query** | **Items found** |
| --- | --- | --- |
| [#1](http://www.ncbi.nlm.nih.gov/pubmed/advanced) | Search "Publication Bias"[Mesh] | [1747](http://www.ncbi.nlm.nih.gov/pubmed/?cmd=HistorySearch&querykey=1) |
| [#2](http://www.ncbi.nlm.nih.gov/pubmed/advanced) | Search "Bias (Epidemiology)"[Mesh] | [44583](http://www.ncbi.nlm.nih.gov/pubmed/?cmd=HistorySearch&querykey=2) |
| [#3](http://www.ncbi.nlm.nih.gov/pubmed/advanced) | Search "Selection Bias"[Mesh] | [3159](http://www.ncbi.nlm.nih.gov/pubmed/?cmd=HistorySearch&querykey=3) |
| [#4](http://www.ncbi.nlm.nih.gov/pubmed/advanced) | Search "Prejudice"[Mesh] | [20912](http://www.ncbi.nlm.nih.gov/pubmed/?cmd=HistorySearch&querykey=4) |
| [#5](http://www.ncbi.nlm.nih.gov/pubmed/advanced) | Search "bias"[tiab] | [64678](http://www.ncbi.nlm.nih.gov/pubmed/?cmd=HistorySearch&querykey=5) |
| [#6](http://www.ncbi.nlm.nih.gov/pubmed/advanced) | Search #1 OR #2 OR #3 OR #4 OR #5 | [122639](http://www.ncbi.nlm.nih.gov/pubmed/?cmd=HistorySearch&querykey=6) |
| [#7](http://www.ncbi.nlm.nih.gov/pubmed/advanced) | Search "Research/ standards"[Mesh] | [19170](http://www.ncbi.nlm.nih.gov/pubmed/?cmd=HistorySearch&querykey=7) |
| [#8](http://www.ncbi.nlm.nih.gov/pubmed/advanced) | Search "Publishing/standards"[Mesh] | [5410](http://www.ncbi.nlm.nih.gov/pubmed/?cmd=HistorySearch&querykey=8) |
| [#9](http://www.ncbi.nlm.nih.gov/pubmed/advanced) | Search "Quality Control"[Mesh] | [36731](http://www.ncbi.nlm.nih.gov/pubmed/?cmd=HistorySearch&querykey=9) |
| [#10](http://www.ncbi.nlm.nih.gov/pubmed/advanced) | Search "Writing/standards"[Mesh] | [1168](http://www.ncbi.nlm.nih.gov/pubmed/?cmd=HistorySearch&querykey=10) |
| [#11](http://www.ncbi.nlm.nih.gov/pubmed/advanced) | Search "Journalism, Medical/ standards"[Mesh] | [584](http://www.ncbi.nlm.nih.gov/pubmed/?cmd=HistorySearch&querykey=11) |
| [#12](http://www.ncbi.nlm.nih.gov/pubmed/advanced) | Search #7 OR #8 OR #9 OR #10 OR #11 | [59526](http://www.ncbi.nlm.nih.gov/pubmed/?cmd=HistorySearch&querykey=12) |
| [#13](http://www.ncbi.nlm.nih.gov/pubmed/advanced) | Search #6 AND #12 | [3044](http://www.ncbi.nlm.nih.gov/pubmed/?cmd=HistorySearch&querykey=13) |
| [#14](http://www.ncbi.nlm.nih.gov/pubmed/advanced) | Search "Registries"[Mesh] | [46060](http://www.ncbi.nlm.nih.gov/pubmed/?cmd=HistorySearch&querykey=14) |
| [#15](http://www.ncbi.nlm.nih.gov/pubmed/advanced) | Search "Evidence-Based Medicine"[Mesh] | [45157](http://www.ncbi.nlm.nih.gov/pubmed/?cmd=HistorySearch&querykey=15) |
| [#16](http://www.ncbi.nlm.nih.gov/pubmed/advanced) | Search "Clinical Trials as Topic"[Mesh] | [250161](http://www.ncbi.nlm.nih.gov/pubmed/?cmd=HistorySearch&querykey=16) |
| [#17](http://www.ncbi.nlm.nih.gov/pubmed/advanced) | Search "Review Literature as Topic"[Mesh] | [6050](http://www.ncbi.nlm.nih.gov/pubmed/?cmd=HistorySearch&querykey=17) |
| [#18](http://www.ncbi.nlm.nih.gov/pubmed/advanced) | Search "Practice Guidelines as Topic"[Mesh] | [65868](http://www.ncbi.nlm.nih.gov/pubmed/?cmd=HistorySearch&querykey=18) |
| [#19](http://www.ncbi.nlm.nih.gov/pubmed/advanced) | Search "Meta-Analysis as Topic"[Mesh] | [11809](http://www.ncbi.nlm.nih.gov/pubmed/?cmd=HistorySearch&querykey=19) |
| [#20](http://www.ncbi.nlm.nih.gov/pubmed/advanced) | Search "Periodicals as Topic"[Mesh] | [31483](http://www.ncbi.nlm.nih.gov/pubmed/?cmd=HistorySearch&querykey=20) |
| [#21](http://www.ncbi.nlm.nih.gov/pubmed/advanced) | Search #14 OR #15 OR #16 OR #17 OR #18 OR #19 OR #20 | [424261](http://www.ncbi.nlm.nih.gov/pubmed/?cmd=HistorySearch&querykey=21) |
| [#22](http://www.ncbi.nlm.nih.gov/pubmed/advanced) | Search #13 AND #21 | [1018](http://www.ncbi.nlm.nih.gov/pubmed/?cmd=HistorySearch&querykey=22) |

EMBASE:

| **No.** | **Query** | **Results** |
| --- | --- | --- |
| #1 | 'bias':ti OR 'bias':ab | **73,191** |
| #2 | 'publication'/exp | **108,591** |
| #3 | 'types of study'/exp | **19,509,350** |
| #4 | 'medical literature'/exp | **113,548** |
| #5 | 'outcome assessment'/exp | **159,312** |
| #6 | #3 OR #4 OR #5 | **19,550,408** |
| #7 | #1 AND #2 AND #6 | **975** |

The Cochrane Library:

| **ID** | **Search** | **Hits** |
| --- | --- | --- |
| #1 | ["Publication Bias"[Mesh]](http://han.donau-uni.ac.at/han/4035/onlinelibrary.wiley.com/o/cochrane/searchHistory?mode=runquery&qnum=1) | 10284 |
| #2 | ["Bias (Epidemiology)"[Mesh]](http://han.donau-uni.ac.at/han/4035/onlinelibrary.wiley.com/o/cochrane/searchHistory?mode=runquery&qnum=2) | 542 |
| #3 | ["Selection Bias"[Mesh]](http://han.donau-uni.ac.at/han/4035/onlinelibrary.wiley.com/o/cochrane/searchHistory?mode=runquery&qnum=3) | 4103 |
| #4 | ["Prejudice"[Mesh]](http://han.donau-uni.ac.at/han/4035/onlinelibrary.wiley.com/o/cochrane/searchHistory?mode=runquery&qnum=4) | 287 |
| #5 | ["bias"[tiab]](http://han.donau-uni.ac.at/han/4035/onlinelibrary.wiley.com/o/cochrane/searchHistory?mode=runquery&qnum=5) | 26095 |
| #6 | [(#1 OR #2 OR #3 OR #4 OR #5)](http://han.donau-uni.ac.at/han/4035/onlinelibrary.wiley.com/o/cochrane/searchHistory?mode=runquery&qnum=6) | 26299 |
| #7 | ["Research/ standards"[Mesh]](http://han.donau-uni.ac.at/han/4035/onlinelibrary.wiley.com/o/cochrane/searchHistory?mode=runquery&qnum=7) | 64 |
| #8 | ["Publishing/standards"[Mesh]](http://han.donau-uni.ac.at/han/4035/onlinelibrary.wiley.com/o/cochrane/searchHistory?mode=runquery&qnum=8) | 11 |
| #9 | ["Quality Control"[Mesh]](http://han.donau-uni.ac.at/han/4035/onlinelibrary.wiley.com/o/cochrane/searchHistory?mode=runquery&qnum=9) | 1090 |
| #10 | ["Writing/standards"[Mesh]](http://han.donau-uni.ac.at/han/4035/onlinelibrary.wiley.com/o/cochrane/searchHistory?mode=runquery&qnum=10) | 6 |
| #11 | ["Journalism, Medical/ standards"[Mesh]](http://han.donau-uni.ac.at/han/4035/onlinelibrary.wiley.com/o/cochrane/searchHistory?mode=runquery&qnum=11) | 1 |
| #12 | [(#7 OR #8 OR #9 OR #10 OR #11)](http://han.donau-uni.ac.at/han/4035/onlinelibrary.wiley.com/o/cochrane/searchHistory?mode=runquery&qnum=12) | 1160 |
| #13 | [(#6 AND #12)](http://han.donau-uni.ac.at/han/4035/onlinelibrary.wiley.com/o/cochrane/searchHistory?mode=runquery&qnum=13) | 254 |
| #14 | ["Registries"[Mesh]](http://han.donau-uni.ac.at/han/4035/onlinelibrary.wiley.com/o/cochrane/searchHistory?mode=runquery&qnum=14) | 1463 |
| #15 | ["Evidence-Based Medicine"[Mesh]](http://han.donau-uni.ac.at/han/4035/onlinelibrary.wiley.com/o/cochrane/searchHistory?mode=runquery&qnum=15) | 3024 |
| #16 | ["Clinical Trials as Topic"[Mesh]](http://han.donau-uni.ac.at/han/4035/onlinelibrary.wiley.com/o/cochrane/searchHistory?mode=runquery&qnum=16) | 35167 |
| #17 | ["Review Literature as Topic"[Mesh]](http://han.donau-uni.ac.at/han/4035/onlinelibrary.wiley.com/o/cochrane/searchHistory?mode=runquery&qnum=17) | 91 |
| #18 | ["Practice Guidelines as Topic"[Mesh]](http://han.donau-uni.ac.at/han/4035/onlinelibrary.wiley.com/o/cochrane/searchHistory?mode=runquery&qnum=18) | 1403 |
| #19 | ["Meta-Analysis as Topic"[Mesh]](http://han.donau-uni.ac.at/han/4035/onlinelibrary.wiley.com/o/cochrane/searchHistory?mode=runquery&qnum=19) | 548 |
| #20 | ["Periodicals as Topic"[Mesh]](http://han.donau-uni.ac.at/han/4035/onlinelibrary.wiley.com/o/cochrane/searchHistory?mode=runquery&qnum=20) | 71 |
| #21 | [(#14 OR #15 OR #16 OR #17 OR #18 OR #19 OR #20)](http://han.donau-uni.ac.at/han/4035/onlinelibrary.wiley.com/o/cochrane/searchHistory?mode=runquery&qnum=21) | 41128 |
| #22 | [(#13 AND #21)](http://han.donau-uni.ac.at/han/4035/onlinelibrary.wiley.com/o/cochrane/searchHistory?mode=runquery&qnum=22) | 57 |

CINAHL, AMED, PsycINFO:

| **#** | **Query** | **Results** |
| --- | --- | --- |
| S11 | S5 or S10 | 604 |
| S10 | S8 and S9 | 85 |
| S9 | S2 or S7 | 101193 |
| S8 | S1 and S6 | 1102 |
| S7 | (MH "Publishing+") | 100879 |
| S6 | (MH "Bias (Research)+") | 6823 |
| S5 | S3 or S4 | 550 |
| S4 | S1 and S2 | 56 |
| S3 | (MH "Publication Bias") | 515 |
| S2 | (DE "PUBLICATIONS") OR (DE "PUBLISHING") | 5208 |
| S1 | TI bias OR AB bias | 43752 |
