# Supplementary material for: Barriers to and facilitators of interventions to counter publication bias: thematic analysis of scholarly articles and stakeholder interviews
Source: BMC Health Serv Res. 2014 Nov 13;14:551. doi: 10.1186/s12913-014-0551-z (PMC4310031; doi:10.1186/s12913-014-0551-z)
Supplement: Additional file 2: — Template for the tentative interview guide. [file 12913_2014_551_MOESM2_ESM.docx]

Additional file 2: Template for the tentative interview guide

**Introduction:**

Thank you so much for your time and for your willingness to participate in this research project. UNCOVER is funded by the European Commission under the 7^th^ framework programme (Grant Number: 282 574) and aims to identify new strategies and ways to counter publication bias in clinical trials.

This interview will focus on identifying strategies to counter publication bias for clinical trials only, and we will explore barriers and facilitators of specific strategies to reduce publication bias.

**Topic 1: Opening question**

I want to start with our working definition of “publication bias”, just to make sure that we are talking about the same phenomenon.

“Publication bias occurs when the publication of research results depends on the nature and direction of the results. Because of publication bias, the results of published studies may be systematically different from those of unpublished studies.“

1. Do you have any comments or questions to this definition?
2. From your perspective, what are major reasons for not publishing the results of clinical trials in the medical field?

**Topic 2: Role of interviewee’s organization and cooperation with other stakeholders**

1. First, can you please describe the aims of *your organization* (and the cooperation with other organizations in the specific field of …)?
2. From your webpage, I have learnt that you have several policies regarding (e.g. research integrity, publication policies, open access policies regarding research reports, articles or raw data, funding policies, …) in place.
   1. What were the major reasons to set up these detailed policies?
   2. What were barriers and facilitators to implement such a guideline within your organization or member organizations?
   3. What kinds of measures (incentives or sanctions) are used to enforce this policy?
3. In general, what can *your organization* do to implement policies and strategies to counter publication bias?

**Topic 3: General questions**

1. From your perspective, what policies and strategies should be implemented to counter publication bias in the field of clinical trials?

**Topic 4: Final questions**

1. Do you have any additional comments to add to our conversation?
