# Supplementary material for: Barriers to and facilitators of interventions to counter publication bias: thematic analysis of scholarly articles and stakeholder interviews
Source: BMC Health Serv Res. 2014 Nov 13;14:551. doi: 10.1186/s12913-014-0551-z (PMC4310031; doi:10.1186/s12913-014-0551-z)
Supplement: Additional file 3: — Types of articles and interventions addressed in thematic analysis for articles. [file 12913_2014_551_MOESM3_ESM.docx]

Additional file 3: Types of articles and interventions addressed in studies included for the review of the scholarly articles

| **Publication** | **Type of article** | **Intervention** |
| --- | --- | --- |
| **Abaid et al., 2007 [35]** | Commentary | - Prospective trial registration - Incentives for reporting |
| **Abaid et al., 2007[36]** | Commentary | - Prospective trial registration |
| **Antonelli & Mercurio, 2009 [29]** | Narrative literature review | - Prospective trial registration - Incentives for reporting |
| **Bock, 2002 [37]** | Commentary | - Changes in peer review and editorial processes |
| **Bonita et al., 2011 [30]** | Narrative literature review | - Prospective trial registration - Incentives for reporting |
| **Bourgeois et al., 2010 [27]** | Empirical Research Study: Analysis of registered trials/ discussion part | - Prospective trial registration - Incentives for reporting |
| **Calnan et al., 2006 [20]** | Empirical Research Study: Expert Interview (n=6) | - Changes in peer review and editorial processes |
| **Chalmers, 2002 [55]** | Letter to the editor | - Incentives for reporting |
| **Connor, 2008 [38]** | Commentary | - Changes in peer review and editorial processes |
| **Deangelis et al., 2005 [46]** | Editorial | - Prospective trial registration |
| **Dickersin & Rennie, 2003 [31]** | Narrative literature review | - Prospective trial registration |
| **Dubben & Beck-Bornholdt, 2005 [34]** | Narrative literature review | - Prospective trial registration |
| **Easterbrook, 1987 [54]** | Letter to the editor | - Incentives for reporting |
| **Gibbs & Wager, 2000 [57]** | Description of a pharmaceutical trial registry | - Prospective trial registration |
| **Glymour, 2005 [53]** | Letter to the editor | - Changes in peer review and editorial processes |
| **Gøtzsche, 2009 [39]** | Commentary | - Public availability of IPD |
| **Gøtzsche, 2011** **[45]** | Commentary | - Public availability of IPD |
| **Hall et al., 2007 [22]** | Empirical Research Study: Analysis of data from protocols submitted to REB/ discussion part | - Prospective trial registration |
| **Henderson, 2002 [40]** | Commentary | - Changes in peer review and editorial processes Public availability of IPD |
| **Joober et al., 2012 [47]** | Editorial | - Incentives for reporting - Changes in peer review and editorial processes |
| **Koletsi et al., 2009 [23]** | Empirical Research Study: Analysis of type of result & impact factor of journals/ discussion part | - Changes in peer review and editorial processes |
| **Laine, 2007 [48]** | Editorial | - Prospective trial registration |
| **Levy, 1992 [41]** | Commentary | - Prospective trial registration - Incentives for reporting |
| **Liesegang, 2009 [42]** | Commentary | - Changes in peer review and editorial processes |
| **Lipworth et al., 2011 [21]** | Empirical Research Study: Expert Interviews (n=35) | - Changes in peer review and editorial processes |
| **McGee et al., 2011 [24]** | Empirical Research Study: Analysis if published trials have been registered/Discussion part | - Prospective trial registration |
| **Newton, 2010 [28]** | Explanatory framework of factors influencing peer review | - Changes in peer review and editorial processes |
| **Phillips, 2011 [49]** | Editorial | - Changes in peer review and editorial processes |
| **Reveiz et al., 2006 [56]** | description of a specific trial registry | - Prospective trial registration |
| **Reynolds, 2003 [50]** | Editorial | - Prospective trial registration - Incentives for reporting |
| **Rising et al., 2008 [25]** | Empirical Research Study: Analysis of registered trials and their publication/ discussion part | - Incentives for reporting |
| **Scherer & Trelle, 2008 [19]** | Empirical Research Study: Web-based survey of academic researchers (n=282) | - Prospective trial registration - Public availability of IPD |
| **Seigel, 2003 [32]** | Narrative literature review | - Peer Review - Disclosure of conflict of commercial interest |
| **Somberg, 2003 [51]** | Editorial | - Prospective trial registration |
| **Staessen, 2003 [44]** | Commentary | - Changes in peer review and editorial processes |
| **Steinbrook, 2004 [43]** | Commentary | - Prospective trial registration - Incentives for reporting |
| **Strech, 2012 [33]** | Narrative literature review | - Prospective trial registration |
| **Tonks, 1999 [52]** | Editorial | - Prospective trial registration |
| **Viergever & Ghersi, 2011 [26]** | Empirical Research Study: Analysis of registered trials/discussion part | - Prospective trial registration |
